# A combined radiomic model distinguishing GISTs from leiomyomas and schwannomas in the stomach based on endoscopic ultrasonography images

**DOI:** 10.1002/acm2.14023

**Published:** 2023-05-11

**Authors:** Xian‐Da Zhang, Ling Zhang, Ting‐Ting Gong, Zhuo‐Ran Wang, Kang‐Li Guo, Jun Li, Yuan Chen, Jian‐Tao Zhang, Ben‐Gong Ye, Jin Ding, Jian‐Wei Zhu, Feng Liu, Duan‐Min Hu, JianGang Chen, Chun‐Hua Zhou, Duo‐Wu Zou

**Affiliations:** ^1^ Department of Gastroenterology, Ruijin Hospital Shanghai Jiao Tong University School of Medicine Shanghai China; ^2^ Shanghai Key Laboratory of Multidimensional Information Processing East China Normal University Shanghai China; ^3^ Department of Gastroenterology The Second Affiliated Hospital of Soochow University Suzhou Jiangsu China; ^4^ Department of Digestive Endoscopy Center Shanghai Tenth People's Hospital, Tongji University School of Medicine Shanghai China; ^5^ Department of Gastroenterology, Jinhua Hospital Zhejiang University School of Medicine Jinhua Zhejiang China; ^6^ Department of Gastroenterology First Hospital of Hanbin District Ankang Shaanxi China

**Keywords:** endoscopic ultrasonography, gastrointestinal stromal tumors, radiomics, stomach, subepithelial lesion

## Abstract

**Background:**

Endoscopic ultrasonography (EUS) is recommended as the best tool for evaluating gastric subepithelial lesions (SELs); nonetheless, it has difficulty distinguishing gastrointestinal stromal tumors (GISTs) from leiomyomas and schwannomas. GISTs have malignant potential, whereas leiomyomas and schwannomas are considered benign.

**Purpose:**

This study aimed to establish a combined radiomic model based on EUS images for distinguishing GISTs from leiomyomas and schwannomas in the stomach.

**Methods:**

EUS images of pathologically confirmed GISTs, leiomyomas, and schwannomas were collected from five centers. Gastric SELs were divided into training and testing datasets based on random split‐sample method (7:3). Radiomic features were extracted from the tumor and muscularis propria regions. Principal component analysis, least absolute shrinkage, and selection operator were used for feature selection. Support vector machine was used to construct radiomic models. Two radiomic models were built: the conventional radiomic model included tumor features alone, whereas the combined radiomic model incorporated features from the tumor and muscularis propria regions.

**Results:**

A total of 3933 EUS images from 485 cases were included. For the differential diagnosis of GISTs from leiomyomas and schwannomas, the accuracy, sensitivity, specificity, and area under the receiver operating characteristic curve were 74.5%, 72.2%, 78.7%, and 0.754, respectively, for the EUS experts; 76.8%, 74.4%, 81.0%, and 0.830, respectively, for the conventional radiomic model; and 90.9%, 91.0%, 90.6%, and 0.953, respectively, for the combined radiomic model. For gastric SELs <20 mm, the accuracy, sensitivity, specificity, and area under the receiver operating characteristic curve of the combined radiomic model were 91.4%, 91.6%, 91.1%, and 0.960, respectively.

**Conclusions:**

We developed and validated a combined radiomic model to distinguish gastric GISTs from leiomyomas and schwannomas. The combined radiomic model showed better diagnostic performance than the conventional radiomic model and could assist EUS experts in non‐invasively diagnosing gastric SELs, particularly gastric SELs <20 mm.

## 1 INTRODUCTION

Gastric subepithelial lesions (SELs) are generally discovered during routine upper endoscopy as intraluminal protuberances covered with normal mucosa.^1^ Gastrointestinal stromal tumors (GISTs) are the most common type of gastric SELs and have a malignant potential.^1^ Endoscopic or surgical resection is recommended for pathologically confirmed GISTs.^2^, ^3^ Differential diagnosis of GISTs from benign gastric SELs, such as gastric leiomyomas and gastric schwannomas, is important to ensure appropriate clinical management.

Endoscopic ultrasonography (EUS) is the best tool for characterizing gastric SELs, including their size, shape, echogenicity, and originating layer.^1^ The gastric wall consists of five layers under EUS with alternating echo intensities. The first, third, and fifth layers are hyperechoic (bright on grayscale images), whereas the second and fourth (muscularis propria [MP]) layers are hypoechoic (dark on grayscale images).^4^ Although EUS can provide important information regarding certain types of gastric SELs, its ability to differentiate gastric SELs originating from the fourth layer, such as GISTs, leiomyomas, and schwannomas, is limited.^5^, ^6^, ^7^ Therefore, a reliable method for distinguishing gastric GISTs from leiomyomas and schwannomas is required.

Radiomic models based on artificial intelligence (AI) have been widely applied in the medical field.^8^, ^9^ By extracting and analyzing quantitative features from medical images, radiomic models can non‐invasively distinguish benign tumors from malignant tumors.^10^, ^11^, ^12^ Miniprobes (MPS), radial scopes, and linear scopes are different types of EUS probes used in gastric SEL evaluation. As the transducer and frequency settings differ between these three types of probes, EUS images accordingly display different characteristics. In previous studies that applied AI models to EUS for gastric SELs diagnosis, the accuracy ranged from 79.2% to 91.2%.^13^, ^14^, ^15^, ^16^, ^17^, ^18^ However, these studies failed to selectively test the performance of AI models using images acquired with MPS, radial scopes, or linear scopes; thus, the universality of the constructed models remains unclear.

Therefore, this pilot study aimed to establish a combined radiomic model based on EUS images to distinguish gastric GISTs from leiomyomas and schwannomas, particularly gastric SELs <20 mm in size. Radiomic features were extracted from the tumor and MP regions. The universality of the combined radiomic model was assessed by subgroup analysis using images acquired by MPS, radial scopes, and linear scopes.

## 2 METHODS

### 2.1 Study population

This study was conducted in accordance with the principles embodied in the Declaration of Helsinki and was approved by the Institutional Review Board of Ruijin Hospital affiliated with Shanghai Jiao Tong University School of Medicine (reference number: 2022031). Owing to the retrospective nature of this study, the requirement for written informed consent was waived. The inclusion criteria were as follows: (1) patients who underwent preoperative EUS for the diagnosis of gastric SELs at Ruijin Hospital affiliated with Shanghai Jiao Tong University School of Medicine, The Second Affiliated Hospital of Soochow University, Tenth People's Hospital of Tongji University, Jinhua People's Hospital, and First Hospital of Hanbin District between January 2012 and June 2022; (2) preoperative EUS examination conducted using MPS, radial scopes, and linear scopes; and (3) pathologically confirmed GISTs, leiomyomas, and schwannomas in the stomach based on surgically or endoscopically resected specimens. The exclusion criteria were as follows: (1) pathological diagnosis based on biopsied specimens, such as EUS‐guided fine‐needle aspiration/biopsy (EUS‐FNA/B) and mucosal incision‐assisted biopsy; (2) poor image quality resulting from blurring, lack of focus, or sonographic artifacts; and (3) inability to identify the MP on EUS images. Bitmap images were used in our study, and images were stored at 24 bits per pixel. Pathological diagnoses made after surgical or endoscopic resection were considered as the golden standard.

Gastric SELs that met the inclusion and exclusion criteria were included and divided into training and testing datasets based on a random split‐sample method (7:3). The “random.sample()”function in Python was used to divide training and testing datasets. In addition, data on demographic and clinical characteristics were collected, including sex, age, lesion size, lesion location, pathological type, GIST risk stratification, and EUS probe used for image acquisition.

### 2.2 EUS procedure

All EUS images were acquired by endoscopists with at least 2 years of experience in EUS procedures using MPS (P2615‐M, P2620‐M: Fujifilm Corporation, Tokyo, Japan; UM‐3R, UM‐DP20‐25R: Olympus Corporation, Tokyo, Japan), radial scopes (EG‐530UR, EG‐580UR: Fujifilm Corporation, Tokyo, Japan; GF‐UM2000: Olympus Corporation, Tokyo, Japan), or linear scopes (EG‐3870U: Pentax Medical, Tokyo, Japan; GF‐UCT180, GF‐UCT260: Olympus Corporation, Tokyo, Japan) and ultrasound systems (SU7000, SU8000, SP900: Fujifilm Corporation, Tokyo, Japan; EU‐ME1, EU‐ME2, EU‐ME2 plus: Olympus Corporation, Tokyo, Japan). The degassed water‐filled method was used to examine gastric wall layers and evaluate specific lesions.^4^ Owing to equipment properties, EUS frequency setting was as follows: MPS at 15−20 MHz, radial scopes at 7.5−12 MHz, and linear scopes at 5−12 MHz. The maximum diameter of the lesions was measured using EUS images.

### 2.3 Segmentation of region of interest

Tumor and muscularis propria (MP) regions were segmented manually by an experienced EUS expert with more than 5 years of experience using LabelMe software (Version 3.0, www.labelme.csail.mit.edu). Region of interest (ROI) segmentation results were evaluated by a senior EUS expert with more than 10 years of experience and adjusted if necessary. Tumor regions were outlined carefully around the outer margins of tumors using the “Create Polygons” option, and MP regions (three to five representative areas) were marked on the same EUS image using the “Create Rectangles” option.

### 2.4 Feature extraction

Two independent sets of radiomic features were constructed. The conventional radiomic model was based on features extracted from the tumor region, whereas the combined radiomic model was based on features extracted from the tumor and MP regions. Pyradiomics (https://www.radiomics.io/pyradiomics.html) was used to extract high‐throughput, quantitative, two‐dimensional features from EUS images. Feature extraction was implemented in the following categories: first‐order features, shape features, gray‐level co‐occurrence matrix (GLCM) features, gray‐level size zone matrix (GLSZM) features, gray‐level run length matrix (GLRLM) features, neighboring gray‐level tone difference matrix (NGTDM) features, and gray‐level dependence matrix (GLDM) features. Moreover, the following features were extracted based on different image types: original, wavelet, Laplacian of Gaussian, square, square root, exponential, logarithm, gradient, and two‐dimensional local binary pattern (LBP2D).

### 2.5 Feature selection and model construction

Principal component analysis (PCA) and least absolute shrinkage and selection operator (LASSO) methods were used to reduce the dimensions of the high‐throughput data. PCA transforms a series of variables with the possibility of linear correlation into a group of linearly unrelated new variables, thus showing the characteristics in a lower dimension. The LASSO regression model predicted and selected the most biologically related features from the EUS image features with nonzero coefficients. Features selected by the PCA and LASSO methods were used for radiomic feature selection. We used Sklearn Package version 0.24.2, Pandas Package version 1.1.5, and Python version 3.6 with Intel Core i7‐11800H as CPU, for all experiments.

Based on the optimal radiomic features, the diagnostic efficiency of four machine learning classifiers was compared in this study: support vector machine (SVM), logistic regression (LR), k‐nearest neighbor, and random forest. These classifiers were applied to the construction of radiomic models using the two sets of features mentioned above. The predictive performance of these four machine learning classifiers was analyzed and evaluated using a receiver operating characteristic curve. The diagnostic efficiency was evaluated using the area under the curve (AUC). The performance of these four classifiers is listed in Table S1. Because SVM achieved the best diagnostic efficiency, it was selected as the classifier for radiomic model construction.

### 2.6 Statistical analysis

The primary outcome was the diagnostic performance of the radiomic models for distinguishing GISTs from non‐GISTs (leiomyomas and schwannomas). The secondary outcome was the diagnostic performance of the EUS experts. Two independent EUS experts with more than 5 years of experience were asked to diagnose EUS images in the testing dataset as GISTs and non‐GISTs. When disagreement occurred, a senior EUS expert with more than 10 years of experience was asked to make the final decision. These experts did not participate in the ROI segmentation and were blinded to pathological diagnosis. Sensitivity, specificity, positive predictive value (PPV), negative predictive value (NPV), accuracy, and AUC were calculated. The sensitivity in our study was defined as the probability of gastric SELs being predicted to be GISTs when gastric SELs were actual GISTs according to pathology. Subgroup analysis was conducted to examine the performance of the combined radiomic model and EUS experts among different EUS probes and lesions of various sizes. Continuous variables with a non‐normal distribution were expressed as medians, first quantiles, and third quantiles, whereas categorical variables are expressed as percentages. The Mann‐Whitney *U* test was used for continuous variables with a non‐normal distribution and ordered categorical variables in paired samples. Pearson's chi‐squared test was used for unordered categorical variables. McNemar's test was used to compare sensitivity and specificity between the conventional radiomic model and the combined radiomic model and between the EUS experts and the combined radiomic model.^19^ Statistical significance was set at *p* < 0.05. All statistical analyses were performed using IBM SPSS Statistics version 27 (IBM Corp., Armonk, NY, USA).

## 3 RESULTS

### 3.1 Demographic and clinical characteristics of patients

**FIGURE 1:**
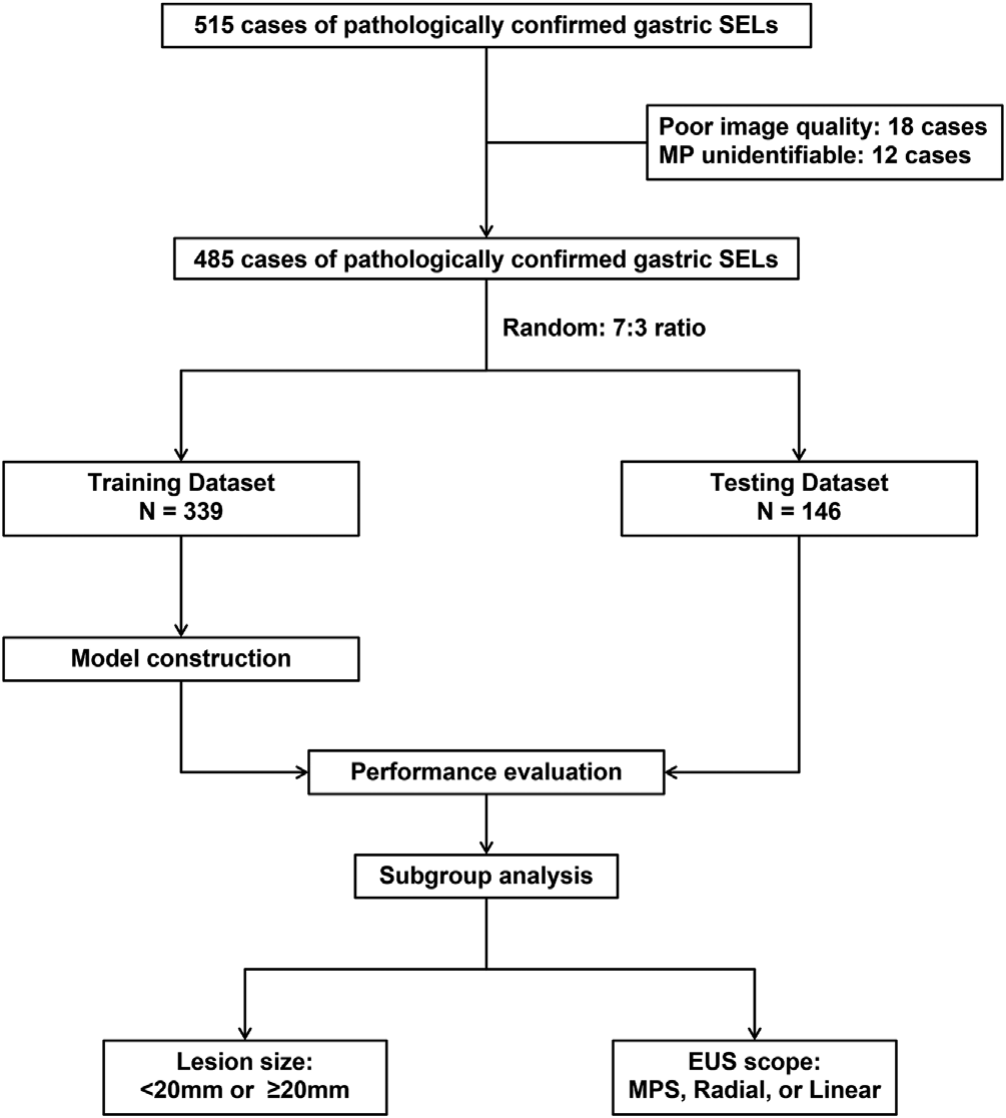
Flowchart of patients and study design. EUS, endoscopic ultrasonography; MP, muscularis propria; MPS, miniprobes; SELs, subepithelial lesions.

**FIGURE 2:**
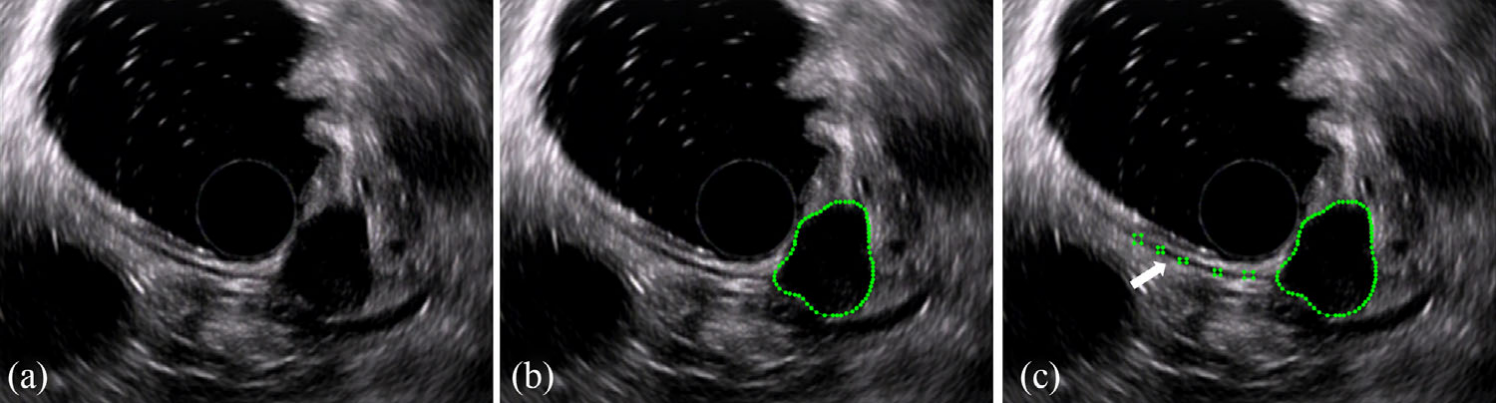
ROI segmentation for radiomic analysis: (a) original EUS image; (b) segmentation of tumor region; and (c) segmentation of tumor region and muscularis propria region (arrow). EUS, endoscopic ultrasonography; ROI, region of interest.

A total of 515 cases of pathologically confirmed gastric SELs were evaluated. Four hundered eighty five cases of gastric SELs meeting the inclusion and exclusion criteria were divided into a training dataset (*N* = 339) and a testing dataset (*N* = 146) (Figure 1). The demographic and clinical characteristics of patients in the training and testing datasets are summarized in Table 1. A total of 2653 EUS images from 208 gastric GIST cases, 107 gastric leiomyoma cases, and 24 gastric schwannoma cases were used as the training dataset, whereas 1280 EUS images from 90 gastric GIST cases, 46 gastric leiomyoma cases, and 10 gastric schwannoma cases were used as the testing dataset. Examples of ROI segmentation on EUS images are shown in Figure 2. There were no significant differences in the patient characteristics between the training and testing datasets.

**TABLE 1:** Demographic and clinical characteristics of patients in the training and testing datasets.

	Training dataset	Testing dataset	
	*N* = 339	*N* = 146	*p*‐value
Sex, *n* (%)			0.744
Male	123 (36.3%)	50 (34.2%)	
Female	216 (63.7%)	96 (65.8%)	
Age, years, median (Q1–Q3)	58.0 (52.0−66.0)	62.0 (54.0−67.0)	0.159
Lesion size, mm, median (Q1–Q3)	14.0 (9.6−24.0)	14.6 (9.2−23.8)	0.651
<20 mm, *n* (%)	222 (65.5%)	96 (65.8%)	1.000
≥20 mm, *n* (%)	117 (34.5%)	50 (34.2%)	
Lesion location, *n* (%)			0.500
Antrum	24 (7.08%)	11 (7.53%)	
Body	161 (47.5%)	74 (50.7%)	
Cardia	41 (12.1%)	22 (15.1%)	
Fundus	113 (33.3%)	39 (26.7%)	
Pathological type, *n* (%)			0.995
GIST	208 (61.4%)	90 (61.6%)	
Leiomyoma	107 (31.6%)	46 (31.5%)	
Schwannoma	24 (7.08%)	10 (6.85%)	
GIST risk stratification, *n* (%)			0.488
Very low	95 (45.7%)	33 (36.7%)	
Low	73 (35.1%)	37 (41.1%)	
Intermediate	25 (12.0%)	14 (15.6%)	
High	15 (7.21%)	6 (6.67%)	
EUS probe, *n* (%)			0.622
MPS	167 (49.3%)	65 (44.5%)	
Radial	156 (46.0%)	74 (50.7%)	
Linear	16 (4.72%)	7 (4.79%)	

Abbreviations: EUS, endoscopic ultrasonography; GIST, gastrointestinal stromal tumor; MPS, miniprobes; Q1, first quantile; Q3, third quantile.

### 3.2 Selection of features and construction of radiomic models

A total of 1409 features were extracted from each EUS image. PCA was used to reduce the dimensions of high‐throughput features. The LASSO regression algorithm was used to select features from the dimension‐reduced radiomic features; the selection process is shown in Figure S1. The selected radiomic features of the conventional radiomic model and combined radiomic model are listed in descending order of importance in Table 2. Based on the selected features, SVM was used to construct the radiomic models.

**TABLE 2:** Selected radiomic features for the conventional radiomic model and combined radiomic model.

Model	Selected radiomic features	Numbers
Conventional radiomic model	Gray‐level emphasis, gray‐level run emphasis, contrast, skewness, kurtosis, mean	6
Combined radiomic model	GLCM_IMC1, gray‐level emphasis, GLCM_MCC, energy, skewness, strength, kurtosis, busyness	8

Abbreviations: GLCM, gray‐level co‐occurrence matrix; IMC, informational measure of correlation; MCC, maximal correlation coefficient.

### 3.3 Performance of the conventional radiomic model, combined radiomic model, and EUS experts in the testing dataset

The testing dataset was used to validate the diagnostic performance of the radiomic models developed using the training dataset. The diagnostic performance of the conventional radiomic model (tumor features alone) and combined radiomic model (tumor features + MP features) are shown in Table 3. By combining tumor features with MP features, the sensitivity improved from 74.4% (conventional radiomic model) to 91.0% (combined radiomic model); additionally, the specificity improved from 81.0% to 90.6%. Differences in sensitivity and specificity between the conventional radiomic model and combined radiomic model were statistically significant (*p* < 0.001). The accuracy also increased from 76.8% to 90.9%. The AUC value of the combined radiomic model was also higher than that of the conventional radiomic model (0.953 vs. 0.830).

**TABLE 3:** Diagnostic performance of the conventional radiomic model and combined radiomic model in the testing dataset.

Model	Sensitivity (%)	Specificity (%)	PPV (%)	NPV (%)	Accuracy (%)	AUC (95% CI)
Conventional radiomic model	74.4	81.0	87.5	63.9	76.8	0.830 (0.801−0.862)
Combined radiomic model	91.0***	90.6***	94.6	84.9	90.9	0.953 (0.933−0.976)

Sensitivity and specificity of the conventional radiomic model and combined radiomic model were compared.

Abbreviations: AUC, area under the curve; NPV, negative predictive value; PPV, positive predictive value.

*** *p* < 0.001.

The diagnostic performance of the EUS experts is presented in Table 4. The accuracy, sensitivity, specificity, and AUC for the EUS experts were 74.5%, 72.2%, 78.7%, and 0.754, respectively. The combined radiomic model fared better accuracy and AUC value than the EUS experts and achieved significantly higher sensitivity and specificity (*p* < 0.001).

**TABLE 4:** Diagnostic performance of EUS experts in the testing dataset.

	Sensitivity (%)	Specificity (%)	PPV (%)	NPV (%)	Accuracy (%)	AUC (95% CI)
Lesion size						
<20 mm	67.0	73.6	82.4	54.8	69.4	0.703 (0.664−0.743)
≥20 mm	79.4	85.1	90.1	70.7	81.5	0.822 (0.785−0.860)
EUS probe						
MPS	74.8	78.6	86.2	63.5	76.1	0.767 (0.714−0.820)
Radial	69.6	79.7	86.1	59.1	73.2	0.748 (0.713−0.784)
Linear	82.1	72.9	83.1	71.4	78.6	0.775 (0.686−0.863)
All	72.2***	78.7***	85.8	61.3	74.5	0.754 (0.726−0.782)

Sensitivity and specificity of the EUS experts and the combined radiomic model were compared.

Abbreviations: EUS, endoscopic ultrasonography; MPS, miniprobes; NPV, negative predictive value; PPV, positive predictive value.

*** *p* < 0.001.

### 3.4 Performance of the combined radiomic model among different types of EUS probes and lesions of various sizes

Because the combined radiomic model showed better diagnostic performance, a subgroup analysis was conducted to test its performance among different types of EUS probes and lesions of various sizes. The diagnostic performance of the combined radiomic model in subgroups of EUS images acquired by MPS, radial scopes, and linear scopes and gastric SELs <20 mm and ≥20 mm in size is presented in Table 5. The accuracy, sensitivity, and specificity of MPS, radial scopes, and linear scopes were all above 90%, except for the specificity of linear scopes (83.3%). For the diagnosis of gastric SELs <20 mm in size, the accuracy, sensitivity, specificity, and AUC value of the combined radiomic model were 91.4%, 91.6%, 91.1%, and 0.960, respectively, whereas those of EUS experts were 69.4%, 67.0%, 73.6%, and 0.703, respectively.

**TABLE 5:** Diagnostic performance of the combined radiomic model in the testing dataset analyzed by subgroups.

	Sensitivity (%)	Specificity (%)	PPV (%)	NPV (%)	Accuracy (%)	AUC (95% CI)
EUS probe						
MPS	91.1***	91.2**	94.9	85.2	91.2	0.948 (0.930−0.967)
Radial	90.3***	91.6***	95.1	83.9	90.8	0.959 (0.943−0.974)
Linear	94.9	83.3	90.2	90.9	90.5	0.891 (0.823−0.959)
Lesion size						
<20 mm	91.6***	91.1***	95.0	85.5	91.4	0.960 (0.944−0.976)
≥20 mm	90.1***	90.0	94.0	84.2	90.1	0.937 (0.917−0.955)

Sensitivity and specificity of the EUS experts and the combined radiomic model were compared.

Abbreviations: AUC, area under the curve; EUS, endoscopic ultrasonography; MPS, miniprobes; NPV, negative predictive value; PPV, positive predictive value.

** *p* < 0.01.

*** *p* < 0.001.

## 4 DISCUSSION

In the present study, we introduced a combined radiomic model for distinguishing gastric GISTs from leiomyomas and schwannomas in the stomach based on EUS images. Radiomic models were developed and evaluated using 485 pathologically confirmed cases of gastric GISTs, leiomyomas, and schwannomas from five centers. Only lesions with a definite pathological diagnosis made after surgical or endoscopic resection were included in our study. Surgical or endoscopic resection is the standard of care for pathologically confirmed GISTs without metastasis.^1^, ^2^ In contrast, because of the low risk of malignant degeneration, excision is only indicated for symptomatic leiomyomas or schwannomas.^1^ The combined radiomic model, which incorporated features extracted from the tumor and MP regions, showed better diagnostic performance than the conventional radiomic model and EUS experts. Further subgroup analysis revealed that the combined radiomic model achieved satisfactory diagnostic performance on images acquired using MPS, radial scopes, and linear scopes. Therefore, the combined radiomic model is expected to effectively and non‐invasively distinguish gastric GISTs from leiomyomas and schwannomas, avoiding unnecessary resection.

Diagnosis of gastric SELs <20 mm in size and originating from the fourth layer of the gastric wall remains challenging for EUS experts. Gastric GISTs, leiomyomas, and schwannomas are seemed as relatively similar hypoechoic to isoechoic tumors originating from the fourth layer of gastric wall.^5^ Thus, it is difficult to distinguish gastric GISTs from leiomyomas and schwannomas. European Society of Gastrointestinal Endoscopy recommends EUS as the best diagnostic tool to identify gastric SELs, however, EUS alone is not sufficient.^1^ Obtaining sufficient tissue for histopathological examination is crucial for diagnosis of gastric SELs; however, the amount of tissue collected is affected by the size of the tumor.^20^ EUS‐FNA is frequently used for tissue sampling in gastric SELs. In cases of gastric SELs <20 mm in size, EUS‐FNA often does not yield sufficient specimens, with adequate sample rates ranging from 35% to 71%.^21^, ^22^, ^23^ EUS‐FNB and mucosal incision‐assisted biopsy have shown superior diagnostic rates to EUS‐FNA; however, the diagnostic rates of each institution varied,^24^, ^25^, ^26^, ^27^, ^28^ partly due to the influence of technical skills. Despite its low incidence, adverse events, such as bleeding and aspiration pneumonia, have been observed with these invasive tissue acquisition methods.^29^, ^30^ For gastric SELs <20 mm in size, the diagnostic accuracy, sensitivity, and specificity of our combined radiomic model were 91.4%, 91.6%, and 91.1%, respectively. The combined radiomic model that we developed may aid EUS experts in non‐invasively diagnosing gastric SELs <20 mm in size.

Recent advances in radiomics have enabled the extraction of high‐throughput quantitative image features beyond what can be seen by the naked eye.^31^ The analysis of image features based on subjective interpretations by EUS performers found few features that could be used to separate gastric GISTs from leiomyomas and schwannomas.^1^, ^5^ Moreover, analysis of the characteristic features of gastric SELs is constrained by poor interobserver agreement resulting from the subjective interpretation of EUS images. Objective quantitative radiomic features extracted from images have been introduced as potential tools to overcome interobserver inconsistency in medical imaging.^32^

Previous studies on the application of AI in EUS have focused on the analysis of tumor features. Kim et al.^17^ reported the usefulness of convolutional neural network (CNN) computer‐aided diagnosis for distinguishing GISTs from non‐GISTs (leiomyomas and schwannomas). This CNN‐based model had a sensitivity, specificity, and accuracy of 83.0%, 75.5%, and 79.2%, respectively, for distinguishing GISTs from non‐GISTs. Minoda et al.^16^ used 173 cases for training and 100 cases for testing and revealed the superior discriminative accuracy for GISTs (86.3% in diameter <20 mm, 90.0% in diameter >20 mm), as compared with that of the EUS experts (73.3% in diameter <20 mm, 53.3% in diameter >20 mm). Yang et al.^13^ investigated the diagnostic ability of a CNN‐based model to differentiate between GISTs and leiomyomas. By using AI, the diagnostic accuracy of EUS experts increased from 73.8% to 88.8%. Interestingly, this study revealed that AI diagnosis of gastric SELs using EUS images depends on the choice of EUS probes.^13^

In our study, we focused on the radiomic features of both the tumor and MP regions and explored the influence of including MP features in radiomic models. EUS images are composed of grayscale pixels, with echogenicity expressed as grayscale values ranging from 0 (anechoic) to 255 (hyperechoic).^33^ The type of EUS probe used and the parameter settings of the equipment (frequency, gain, brightness, contrast, acoustic power, and slope time) varied among procedures; thus, the tumor region may display different EUS image characteristics correspondingly.^33^, ^34^ MPS, radial scopes, and linear scopes are different kinds of EUS probes used in gastric SEL evaluation. Ultrasound waves are produced and detected using transducers. MPSs are typically equipped with mechanical transducers, whereas radial scopes and linear scopes are equipped with electronic transducers. Owing to the functional differences between mechanical transducers and electronic transducers, EUS images acquired using MPS, radial scopes, and linear scopes have different characteristics.^35^

To overcome this, we incorporated the features of the MP in our model. Echogenicity of the MP is often referred to as the standard of hypoechogenicity in examinations. Iwamuro et al.^36^ used echogenicity of the MP to standardize the absolute echogenicity of the tumor to objectively evaluate the lesion. This study revealed that leiomyomas showed echogenicity similar to that of the MP, whereas the echogenicity of the granular cell tumors was close to that of the submucosal layer. Kim et al.^37^ and Lee et al.^38^ selected the echogenicity of the anechoic center and outer hyperechoic rim of the EUS scope to standardize EUS images. After standardization, the mean echogenicity values and the standard deviation of echogenicity in the tumors were analyzed to distinguish GISTs from leiomyomas and schwannomas.^37^, ^38^ The radiomic features in our combined radiomic model, such as GLCM_IMC1 and GLCM_MCC, have integrated characteristics extracted from the tumor and MP regions. These features revealed a correlation between tumor and MP characteristics, including the intensity and variation of echogenicity. We expected that the inclusion of MP features could improve the universality of our combined radiomic model, which is applicable in different centers using MPS, radial probes, and linear probes.

Despite the importance of the combined radiomic model, this study had several limitations. First, a combined radiomic model was used for the differential diagnosis of GISTs from non‐GISTs (leiomyomas and schwannomas) in the stomach. Glomus tumors, discovered as hypo‐ or hyperechoic masses arising from the fourth layer, can mimic GISTs.^39^ Glomus tumors make up less than 1% of gastric SELs.^40^ Due to the low incidence, cases of glomus tumors in our centers were limited, so we were unable to include glomus tumors in the current study. Therefore, it is necessary to include glomus tumors in future studies. Other types of gastric SELs, such as lipomas and lymphangiomas, were not included in the current study because their EUS features were distinct from those of GISTs and can be recognized by EUS performers. Second, subgroup analysis of the types of EUS probes was limited to three categories of MPS, radial probes, and linear probes, instead of specific products from different manufacturers. EUS probes and systems from various manufacturers use different techniques for signal attenuation compensation and different algorithms for EUS radio frequency signal processing. These might bias the extraction of tumor features in our combined radiomic model. We attempted to sub‐analyze EUS probes based on various products; however, the small sample size of each subgroup affected the credibility of the results. This finding might lessen the generalizability of our combined radiomic model under EUS probes and systems from different manufacturers. Third, frequency setting and post‐processing might limit the robustness of our model. In clinical practice, different frequencies are used by EUS performers to better characterize tumor features. Post‐processing enhances image quality of ultrasound scanners by reducing noise and enhancing contrast. Unfortunately, post‐processing algorithms are variable across manufacturers and are generally kept proprietary as a black box to the user.^41^ These difference in image acquisition and processing might cause variability in image features, affecting the performance of ultrasound‐based machine learning models.^42^ Deep learning‐based image conversion could improve the reproducibility of radiomic features under technical variation.^43^ Fourth, the retrospective nature of the study may have led to a selection bias. Thus, further large‐scale, multicenter prospective studies are required to confirm the universality and diagnostic performance of our combined radiomic model.

## 5 CONCLUSIONS

We developed and validated a combined radiomic model for distinguishing GISTs from leiomyomas and schwannomas in the stomach, based on features extracted from the tumor and MP regions on EUS images. The model showed desirable predictive performance and may assist EUS experts in non‐invasively diagnosing gastric SELs, particularly gastric SELs <20 mm in size.

## AUTHOR CONTRIBUTIONS

Xian‐Da Zhang, Kang‐Li Guo, Jun Li, Yuan Chen, Jian‐Tao Zhang, Ben‐Gong Ye, Jin Ding, Jian‐Wei Zhu, Feng Liu, and Duan‐Min Hu collected the data. Xian‐Da Zhang, Ling Zhang, and Ting‐Ting Gong prepared the materials. Zhuo‐Ran Wang and Jian‐Gang Chen performed data analysis. Chun‐Hua Zhou and Duo‐Wu Zou contributed to the study conception and design. Xian‐Da Zhang, Ling Zhang, and Ting‐Ting Gong wrote the first draft of the manuscript. All authors commented on the previous versions of the manuscript. All authors read and approved the final manuscript.

## CONFLICT OF INTEREST STATEMENT

The authors have no relevant conflicts of interest to disclose.

## ETHICS APPROVAL

This study was approved by the Institutional Review Board of Ruijin Hospital affiliated with Shanghai Jiao Tong University School of Medicine (reference number: 2022031) and conducted according to the Helsinki Declaration.

## Supporting information

Supporting Information

Supporting Information

## Data Availability

The data used to support the current study are available from the corresponding authors upon reasonable request.
