# Supplementary material for: A combined radiomic model distinguishing GISTs from leiomyomas and schwannomas in the stomach based on endoscopic ultrasonography images
Source: J Appl Clin Med Phys. 2023 May 11;24(7):e14023. doi: 10.1002/acm2.14023 (PMC10338752; doi:10.1002/acm2.14023)
Supplement: Supplementary file 1 — Supporting Information [file ACM2-24-e14023-s001.docx]

**SUPPLEMENTARY MATERIALS**

**SUPPLEMENTARY TABLE S1** Diagnostic performance of four machine learning classifiers in the testing datasets.

| **Model** | **Classifier** | **Testing dataset (*N*=146)** | | | |
| --- | --- | --- | --- | --- | --- |
|  |  | **Sensitivity (%)** | **Specificity**  **(%)** | **Accuracy (%)** | **AUC (95% CI)** |
| Conventional radiomic model | SVM | 74.4% | 81.0% | 76.8% | 0.830 (0.801–0.862) |
|  | LR | 66.3% | 55.1% | 62.3% | 0.648 (0.592–0.691) |
|  | KNN | 75.3% | 68.4% | 72.8% | 0.759 (0.726–0.799) |
|  | RF | 80.4% | 46.0% | 68.0% | 0.751 (0.709–0.794) |
| Combined radiomic model | SVM | 91.0% | 90.6% | 90.9% | 0.953 (0.933–0.976) |
|  | LR | 60.9% | 67.3% | 63,2% | 0.676 (0.628–0.723) |
|  | KNN | 93.2% | 88.4% | 91.5% | 0.951 (0.932–0.967) |
|  | RF | 82.7% | 59.9% | 74.5% | 0.828 (0.801–0.865) |

Abbreviations: AUC, area under the curve; CI, confidence interval; SVM, support vector machine; LR, logistic regression; KNN, k-nearest neighbor; RF, random forest.


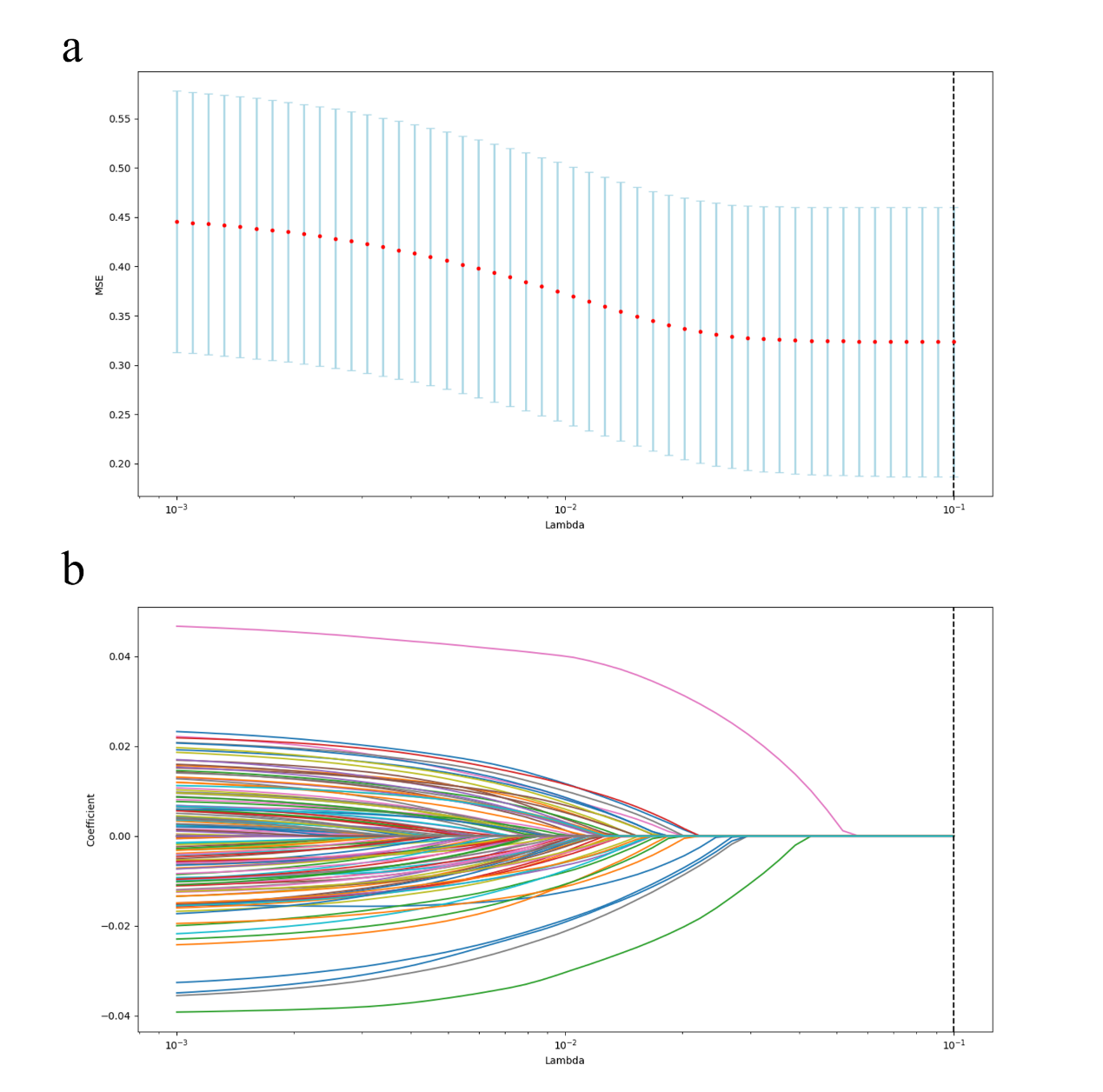


**SUPPLEMENTARY FIGURE S1** Radiomic feature selection using the LASSO logistic regression model. (a) Selection of tuning parameters (lambda) in the LASSO logistic regression model with five-fold cross-validation. (b) LASSO coefficient distribution of radiomic features.
