# Supplementary figures and images for: A combined radiomic model distinguishing GISTs from leiomyomas and schwannomas in the stomach based on endoscopic ultrasonography images
Source: J Appl Clin Med Phys. 2023 May 11;24(7):e14023. doi: 10.1002/acm2.14023 (PMC10338752; doi:10.1002/acm2.14023)

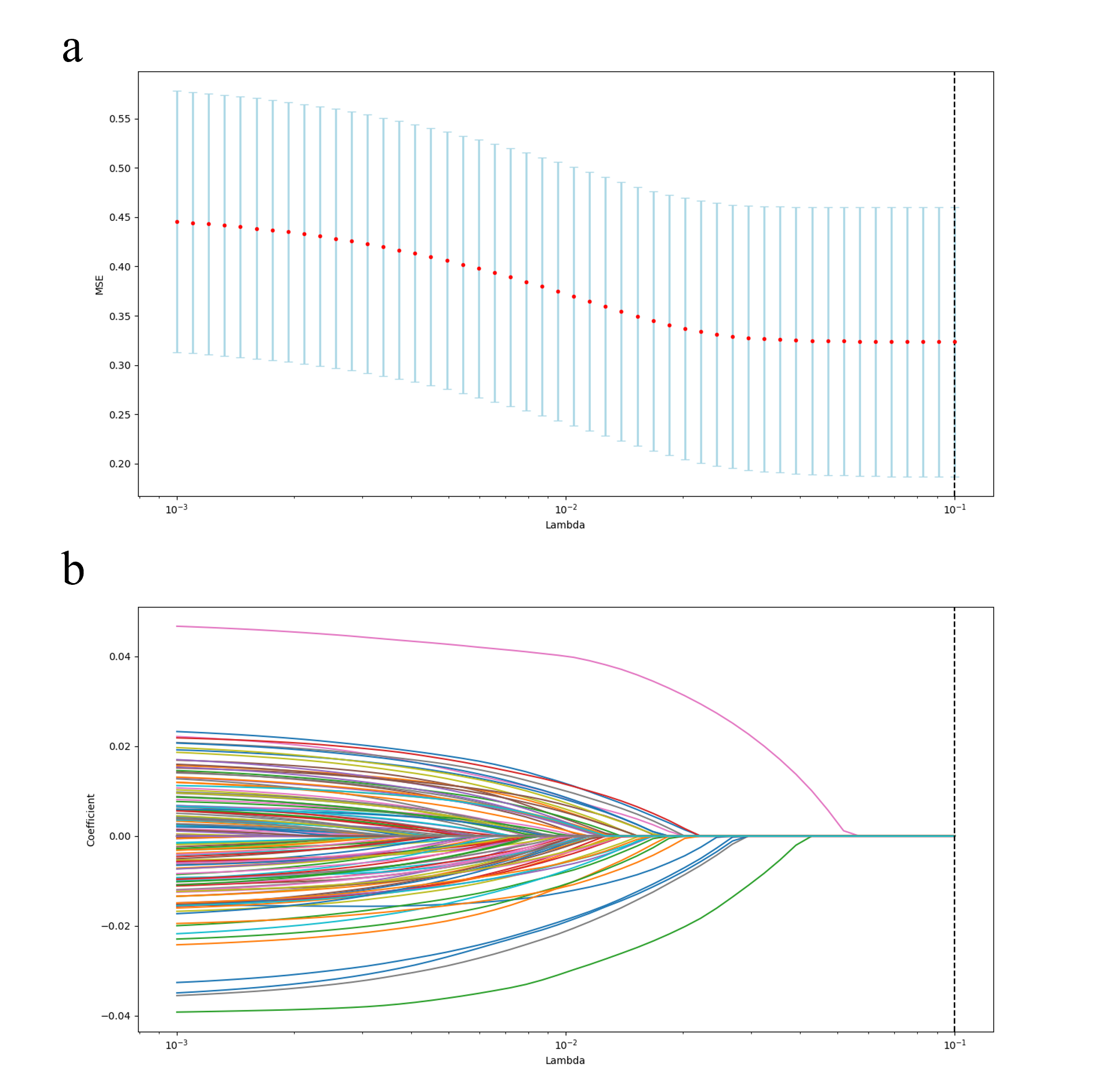

Supplement: Supplementary file 2 — Supporting Information [file ACM2-24-e14023-s002.tif]
